# An incentive-based mitigation strategy to encourage coexistence of large mammals and humans along the foothills of Indian Western Himalayas

**DOI:** 10.1038/s41598-021-84119-7

**Published:** 2021-03-04

**Authors:** Ruchi Badola, Tanveer Ahmed, Amanat Kaur Gill, Pariva Dobriyal, Goura Chandra Das, Srishti Badola, Syed Ainul Hussain

**Affiliations:** Wildlife Institute of India, Chandrabani, Post Box # 18, Dehradun, Uttarakhand 248001 India

**Keywords:** Ecosystem services, Environmental economics

## Abstract

Escalation of human–wildlife conflict (HWC) is a barrier to the conservation of ecological corridors across the globe. The existing mechanisms to counter HWC are either economically and socially taxing, or ineffective for long-term management. We assessed HWC in the corridor linking the Rajaji and Corbett Tiger Reserves in Uttarakhand, India, and its drivers, along with the benefits derived by local communities from the forest. We designed an innovative incentive-based mitigation mechanism to encourage coexistence of people and wildlife around the corridor. Costs incurred due to conflict and benefits derived from the forest were assessed using semi-structured questionnaire-based personal interviews (n = 757) with representatives from forest dependent households (hh). Fuelwood (1678.7 ± 131 kg hh^−1^ year^−1^), fodder (4772 ± 186 kg hh^−1^ year^−1^) and green/dry grass (3359 ± 104 kg hh^−1^ year^−1^) contributed 3 ± 1%, 6 ± 0.5% and 9 ± 1%, respectively, to the annual income of dependent households. 69% of the households practising agriculture reported crop damage by wild animals, 19% of the households that owned livestock reported livestock loss, and 1.58% reported attack on humans resulting in injuries. The cost incurred due to crop raiding and livestock depredation was US $ 159.83 ± 1.0 hh^−1^ year^−1^ and US $ 229.32 ± 34.0 hh^−1^ year^−1^, respectively. Crop loss was positively associated with the number of crops grown per season and cultivation of sugarcane, wheat and pulses, and negatively with distance from forest and cultivation of fodder and finger millet. Livestock depredation was negatively associated with distance from forest and positively with number of livestock owned, primarily calves. The accounting profit from cultivating a hectare of land, in the absence of crop depredation by wild animals, was estimated at US $ 3571.84 ha^−1^ year^−1^ and US $ 361.44 ha^−1^ year^−1^ for the plains and hills, respectively. This value can be used to calculate the payments to be paid to local communities to encourage them to adopt HWC resistant agricultural and pastoralism practices. The net present value of benefits from participating in the payments to encourage coexistence programme for 5 years, discounted at 12%, was US $ 12,875.7 ha^−1^ for the plains and US $ 1302.9 ha^−1^ for the hills.

## Introduction

The burgeoning human population, coupled with agricultural expansion and unplanned rapid infrastructure development, has fragmented natural ecosystems^1^. Fragmentation alters the composition and configuration of a landscape and impacts the biodiversity^2^. Though the importance of patches and isolation due to fragmentation is highlighted by the island biogeography theory^3^, the application of this theory to the terrestrial environment is questionable. Meta-population theory suggests that isolated patches may not support populations of species individually; there should be a certain exchange of individuals and genetic material among the populations^4^. Thus, corridors become a significant factor in terrestrial conservation. Forest corridors facilitate shifting of species in response to climate change, movement of individuals, and provide several direct and indirect ecosystem services vital for human wellbeing^5–10^.

In India, most of the corridors connecting large forest patches including protected areas (PAs), are shared by wild animals and people, mainly natural resource-dependent local communities^11,12^. These corridors are under immense pressure due to increasing human population, high demand for land for human settlements within the corridors and biotic pressures like livestock grazing, extraction of various forest products, mining activities and various development projects^11,12^. The degradation of habitats, coupled with competition for limited resources, forces wild animals into human settlements and their vicinity, leading to human–wildlife conflict (HWC) in the form of crop raiding, livestock depredation and human injuries and deaths, ultimately threatening the survival of the wild animals^13,14^.

The economic and social losses due to human injuries and casualties, crop damage and livestock depredation lead to hostility and reduced support for wildlife conservation by local communities. Local people respond to conflict by poisoning, shooting or trapping wild animals, which counteract conservation measures undertaken by wildlife managers^15^. The existing mechanisms available for mitigating HWC include fencing, translocation and sterilization. These mechanisms are more taxing, both monetarily and physically, and are ineffective in long-term management^16,17^. The compensatory schemes in third world countries like India have largely failed to compensate the incurred losses^18–20^. Thus, there is a need to find innovative measures to effectively reduce conflict and create conditions for peaceful coexistence of humans and wild animals.

Incentive-based mechanisms have been shown to be more effective compared to compensatory schemes^21^. Direct payments or incentives, considered a form of payment for ecosystem services (PES)^22,23^, as an effective conservation tool are gaining the approval of the scientific community and increasingly being implemented to garner the support of local people for conservation actions^24^. PES is implemented by establishing appropriate prices and giving financial incentives to landowners in exchange for activities that support conservation initiatives^25^. Wunder^26^ defined PES as “*a voluntary transaction where a well-defined ecosystem service (or a land use likely to secure that service) is “bought” by a (minimum of one) ecosystem service buyer from a (minimum of one) ecosystem service provider; if and only if the service provider secures ecosystem service provision (conditionality)*”*.* This widely quoted definition highlights the five requirements of PES: (a) voluntary transaction, (b) well-defined ecosystem service that is traded, (c) ecosystem service buyer, (d) ecosystem service seller or natural resource custodian, and (e) payment conditional upon provision of ecosystem service or land use practice. By creating a market for the concerned ecosystem service, PES is primarily implemented with respect to a particular goal such as protection or management of natural resources of national importance, or to address depletion in resources noticed by at least one stakeholder group^27^. As long as the benefit is greater than the cost of conservation, support of local communities and other stakeholders for such initiatives will not be compromised^28^.

In the present study, we assessed HWC and its drivers in the corridor linking the Rajaji and Corbett Tiger Reserves (TRs) located in the foothills of Western Himalayas, along with the cost incurred due to crop raiding and livestock depredation. We also evaluated the monetary contribution of the forest resources to the economy of forest-dependent communities. To promote coexistence of humans and wildlife, we designed an incentive-based mechanism on the principles of PES to make people proactive in conservation actions.

## Results

### Household (hh) characteristics

Of the total households sampled (n = 757), 52% were male and 48% were female. The households had an average family size of 5.2 ± 0.08 individuals hh^−1^, with an average landholding of 1.00 ± 0.07 ha hh^−1^. Households in the hills had a comparatively smaller average family size (4.8 ± 0.1 hh^−1^) and landholding (0.38 ± 0.04 ha hh^−1^) than the plains (Table 1). Only 17% of the household members were illiterate while the rest had received a formal education up to the eighth grade or more (Table 1). Comparatively, more people in the plains were educated up to the eighth grade or more than in the hills (Table 1). Households used a combination of wood, liquefied petroleum gas (LPG), kerosene, biogas and cow dung cakes, and only 20% of the households were solely dependent on wood for fuel (Table S1). Wood was the major source of fuel for 90.6% of the households (98.19% in hills; 88.4% in plains) and was supplemented by LPG (67.7%) and others sources (Table 1). The proportion of households using LPG was higher in the plains (72%) (Table 1). The average consumption of LPG per household was 0.9 ± 0.03 cylinders per month; lower in the hills (0.21 ± 0.02 cylinders per month) than in the plains (0.8 ± 0.04 cylinders per month).

**Table 1:** Characteristics of respondents’ households in the forest corridor linking the Rajaji and Corbett Tiger Reserves, Uttarakhand, India. (* values in parentheses indicate percentage of total households sampled).

Characteristic	Household details		
	Hill	Plain	Overall
Number of villages sampled	14	23	37
Number of households sampled	166	591	757
Household size	4.8 ± 0.1	5.29 ± 0.10	5.2 ± 0.08
Sex ratio (male:female)	1:0.88	1:0.85	1:0.85
Average age of respondents	33.14	30	30.72
**Respondent education (%)**			
Illiterate	50.9	20.3	17.0
Less than 8th grade	10.5	16.9	19.8
8th grade to 12th grade	35.0	48.1	50.3
Graduate	1.4	8.0	6.7
Postgraduate	2.2	6.8	6.1
Average landholding (ha hh^−1^)	0.38 ± 0.04	1.17 ± 0.95	1.00 ± 0.07
**Source of fuel (%)**			
LPG*	52.4 (26.6)	72.0 (41.5)	67.7
Kerosene*	42.7 (21.7)	6.2 (3.6)	14.2
Dung*	0.0	2.4 (1.3)	1.8
Wood*	98.1 (50.0)	88.4 (50.9)	90.6
Biogas*	3.0 (1.5)	4.4 (2.5)	4.0
Households practicing agriculture	152 (91.5%)	382 (64.4%)	534 (70.5%)
Annual income from crops (US $)	212.29 ± 36.1	1069.37 ± 588.5	881.51 ± 19.0
Households with livestock	157 (94.5%)	490 (82.9%)	647 (85.4%)
Average livestock (hh^−1^)	4.58 ± 0.34	5.43 ± 0.21	5.25 ± 0.18
Annual income from livestock (US $ hh^−1^)	55.78 ± 13.5	107.21 ± 22.8	95.93 ± 17.5
Annual income from off-farm sources (US $ hh^−1^)	1918.39 ± 171.0	1969.81 ± 109.5	1958.57 ± 93.3
Total annual income excluding forest contribution (US $ hh^−1^)	1980.67 ± 176.0	2983.92 ± 484.6	2763.91 ± 377.8

54.3% of the households relied on a single source of livelihood, 36.5% relied on two sources and 7.4% were engaged in more than two sources (Fig. S1, Table S2). Agriculture was the mainstay of 70% of the households with crop cultivation providing an income of US $ 881.51 ± 19.0 year^−1^. Most of the households (85.4%) supplemented their income by rearing livestock. The average livestock owned per household was 5.25 ± 0.18, which provided an income of US $ 95.9 ± 17.5 year^−1^. Households also earned their livelihood through private jobs (14%), labour (14%), pension in case of retired government employees (8.9%), business (8.5%), government jobs (8.5%), and horticulture (0.09%). Off-farm activities provided an average annual income of US $ 1958.57 ± 93.3 year^−1^. The average total income of household from all the sources (excluding forest resources income) was US $ 2763.91 ± 377.8 year^−1^ (Table 1).

Households practising agriculture (91.5%) and rearing livestock (94.5%) was higher in the hills than the plains (Table 1). Households in the hills mainly grew finger millet or ragi (*Eleusine coracana*) and vegetables, while wheat (*Triticum* spp.), rice (*Oryza sativa*) and sugarcane (*Saccharum officinarum*) were the main crops grown in the plains. The average number of livestock owned in the hills and plains was 4.58 ± 0.34 hh^−1^ and 5.43 ± 0.21 hh^−1^, respectively. The average income generated from crop cultivation and livestock rearing was lower in hills than the plains. The average income (excluding forest resources) of the households was comparably lower in the hills (US $ 1980.67 ± 176.0 hh^−1^ year^−1^) than in the plains (US $ 2983.92 ± 484.6 hh^−1^ year^−1^; Table 1).

### Benefits of living around the corridor

Of the households surveyed, 84% supplemented their livelihood from forest resources viz., fuelwood (70.4%), fodder (27.5%) and green/dry grass (19.7%). Comparatively, a higher proportion of households in the hills (96%) were dependent on forest resources than the plains (80%). Extraction of forest products, specifically fuelwood (1678.7 ± 131 kg hh^−1^ year^−1^), fodder (4772 ± 186 kg hh^−1^ year^−1^) and green/dry grass (3359 ± 104 kg hh^−1^ year^−1^), contributed 3 ± 1%, 6 ± 0.5% and 9 ± 1%, respectively, to the average annual household income (Table 2). The contribution of forest products to the household income ranged from 6 ± 0.7% (fuelwood) to 12 ± 0.7% (grass) in the hills, and 3 ± 0.7 % (fuelwood) to 8 ± 0.3% (grass) in the plains (Table 2).

**Table 2:** Benefits derived from the forest corridor linking the Rajaji and Corbett Tiger Reserves, Uttarakhand, India. (* values in parentheses indicate percentage of total households sampled)

Resource	Household dependent on forest resources (%)			Extraction (kg hh^−1^ year^−1^) Mean ± S.E			Benefit (US $ hh^−1^ year^−1^) Mean ± S.E			Contribution of forest product (% hh^−1^ year^−1^) Mean ± S.E		
	Hills*	Plains*	Overall	Hills	Plains	Overall	Hills	Plains	Overall	Hills	Plains	Overall
Fuelwood	90.4 (19.8)	64.6 (50.5)	70.4	2304 ± 106	1433 ± 176	1678.7 ± 131	122 ± 5.6	76 ± 9	89 ± 7	6 ± 0.7	3 ± 0.7	3 ± 1
Fodder	85.5 (18.7)	11.2 (8.7)	27.5	4904 ± 233	4486 ± 302	4772 ± 186	259 ± 12	237 ± 16	252 ± 10	9 ± 0.3	4 ± 0.1	6 ± 0.5
Grass	68.1 (14.9)	6.1 (4.7)	19.7	3705 ± 87	2273 ± 266	3359 ± 104	196 ± 5	120 ± 14	177 ± 5	12 ± 0.7	8 ± 0.3	9 ± 1

### Conflict and cost of living around the corridor

**Figure 1:**
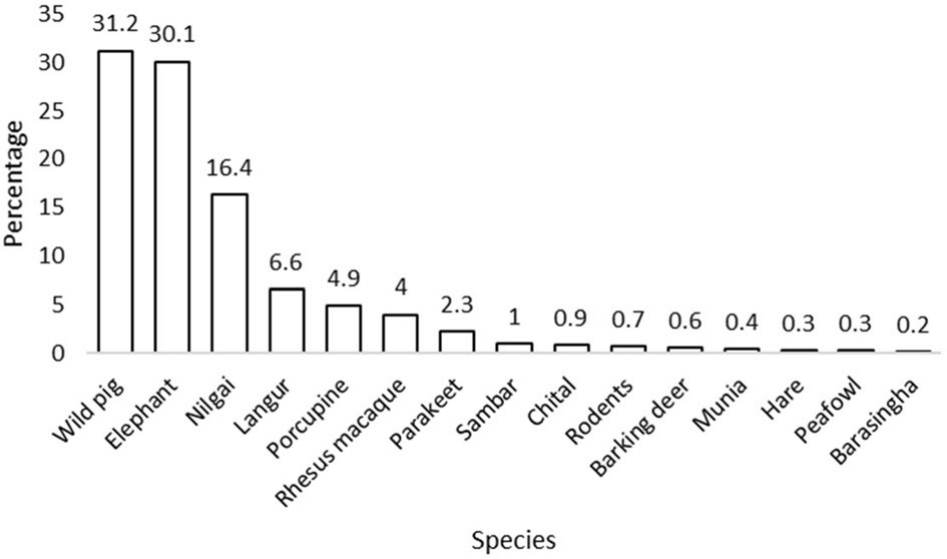
Percentage of respondent reported wild animals involved in crop raiding around the forest corridor linking the Rajaji and Corbett Tiger Reserves, Uttarakhand, India.

Around 69% of the households practicing agriculture (n = 534) reported crop damage by wildlife (Table 3). Incidents of crop raiding were higher in the hills (94.7%) than in the plains (Table 3). A total of 15 species of wildlife, comprising of eight herbivores, two primates, three birds, one lagomorph and one rodent, were reportedly involved in crop damage. More than half of the respondents reported that wild pig (31.2%) and Asian elephant (30.1%) were the most problematic species in the region (Fig. 1). Rice fields were raided the most (0.44 ± 0.02 ha hh^−1^), along with wheat (0.09 ± 0.02 ha hh^−1^) and sugarcane (0.004 ± 0.00 ha hh^−1^; Table S4).

**Table 3:** Cost incurred due to wildlife around the forest corridor linking the Rajaji and Corbett Tiger Reserves, Uttarakhand, India. (* values in parentheses indicate percentage of total households sampled)

Costs incurred	Hills	Plains	Overall
Households practicing agriculture	152	382	534
Households owning livestock	157	490	647
Households that reported crop raiding	144 (94.74%)	224 (58.64%)	368 (68.91%)
Households that reported livestock depredation	29 (18.47%)	94 (19.18%)	123 (19.01%)
Losses from crop raiding (US $ hh^−1^ year^−1^)	69 ± 9.7	325.91 ± 29.5	159.83 ± 1.0
Losses from livestock depredation (US $ hh^−1^ year^−1^)	112.89 ± 21.9	174.31 ± 44.3	229.32 ± 34.0
Total losses due to crops raiding and livestock depredation (US $ hh^−1^ year^−1^)	77.11 ± 9.4	352.78 ± 27.5	257.62 ± 18.9
Total losses (% of total income)	3.61	11.61	9.07

**Figure 2:**
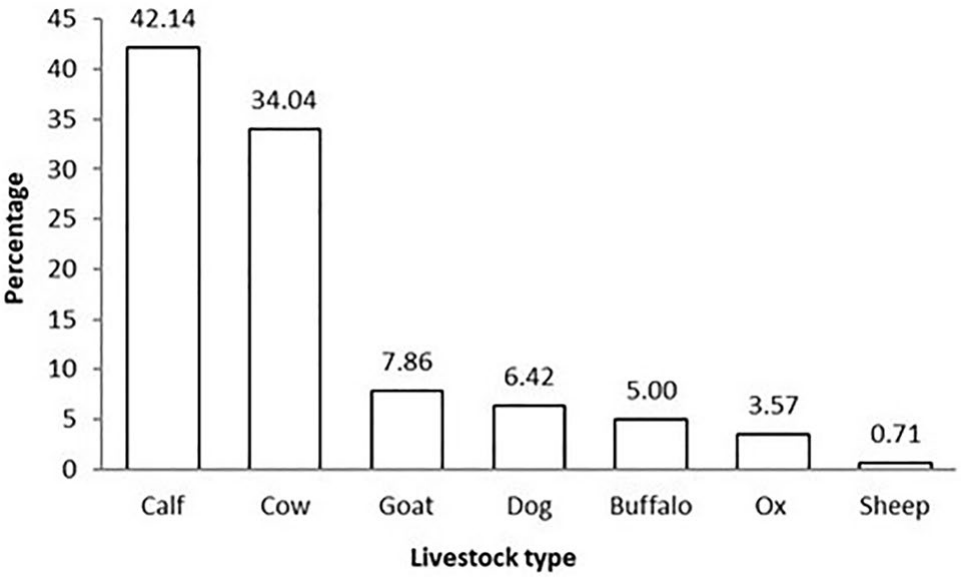
Percentage of respondent reported depredation of different livestock types, including dogs, around the forest corridor linking the Rajaji and Corbett Tiger Reserves, Uttarakhand, India.

Around 19% of the households that owned livestock (n = 647) reported livestock depredation, of which 81.4% attributed the incidents to leopards and 17.1% to tigers. Calves (42.1%) and adult cows (34%) were the victims of these large carnivores most frequently (Fig. 2). Livestock depredation mostly occurred inside the forest (75%), while about 25% occurred in residential areas. The incidents of livestock depredation was almost equal in the hills and the plains (Table 3). Cases of human injury/attacks (1.58% of hh) were also reported.

**Figure 3:**
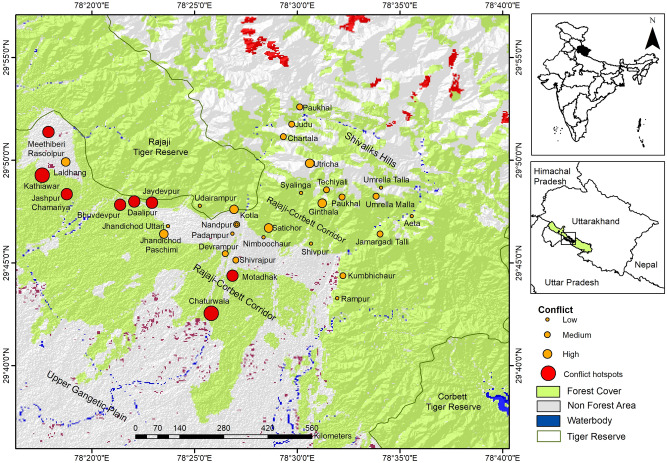
Villages identified as conflict hotspots based on average cost incurred (US $ hh^−1^ year^−1^) due to livestock depredation and crop raiding around the forest corridor linking the Rajaji and Corbett Tiger Reserves, Uttarakhand, India. Land cover used was downloaded from Diva-Gis (http://www.diva-gis.org/gdata). The map was created using ArcGIS v.10.3.1 software developed by ESRI (https://www.esri.com).

The respondents incurred average costs of US $ 159.83 ± 1.0 hh^−1^ year^−1^ and US $ 229.32 ± 34 hh^−1^ year^−1^ due to crop raiding and livestock depredation, respectively (Table 3). The costs incurred due to crop raiding and livestock depredation were greater in the plains (Table 3). The overall cost of wildlife conflict in the hills (US $ 77.11 ± 9.4 hh^−1^ year^−1^) and plains (US $ 352.78 ± 27.5 hh^−1^ year^−1^) reduced the income of the respondents by 3.6% and 11.6%, respectively (Table 3). All hotspots of conflict were located in the western and central-southern part of the corridor, with villages Judu and Aeta in the hills and villages Daalipur and Laldhang in the plains identified as hotspots for livestock depredation, and villages Rasoolpur, Meethiberi, Katiawar, Bhuvdevpur, Jaydevpur, Daalipur and Motadhak in the plains identified as hotspots for crop raiding (Figs. 3, S2, S3).

### Factors governing human–wildlife conflict

The receiver operating characteristic (ROC) curves for crop raiding and livestock depredation were 0.82 ± 0.01 (95%) and 0.73 ± 0.02 (95%), respectively, indicating good fits of regression. Crop loss was positively associated with number of crops grown per season and cultivation of sugarcane, wheat and pulses, while the association was negative with distance from the forest and cultivation of fodder and finger millet (Table 4). The likelihood that livestock depredation will occur increased with increasing number of indigenous buffaloes and calves (Table 4).

**Table 4:** Coefficients (β) for predicting crop damage, livestock depredation and positive attitude of local communities towards wildlife around the forest corridor linking the Rajaji and Corbett Tiger Reserves, Uttarakhand, India.

Predictor	Crop loss		Livestock loss		Attitude		
	B (S.E.)	Wald	B (S.E.)	Wald	B (S.E.)		Wald
Constant	− 0.128*** (0.091)	1.963	− 1.734*** (0.105)	271.06	0.218 (0.236)		0.855
Distance from forest	− 0.411*** (0.097)	17.96					
Wheat	0.457*** (0.114)	16.13					
Finger millet (ragi)	− 0.393** (0.137)	8.21					
Fodder	− 0.341** (0.099)	11.91					
Sugarcane	0.244** (0.105)	5.41					
Pulses	0.364** (0.100)	13.31					
Number of crops cultivated	0.785*** (0.139)	32.09					
Indigenous Buffalo			0.0.335*** (0.079)	17.71			
Calf			0.417*** (0.097)	18.45			
Increase in wildlife population					− 0.479*** (0.153)		9.80
Location (plains)					0.866*** (0.182)		22.76
Chi square	6.33**		16.27		9.87		
Log likelihood	772.612		616.57		997.84		
Nagelkerke’s R^2^	0.406		0.089		0.054		
Hosmer and Lemeshow test	0.44		0.00		0.243		
ROC	0.826 ± 0.01		0.73 ± 0.02		0.62 ± 0.02		

*, ** and *** indicate significance at levels of 0.5, 0.01 and 0.001, respectively.

### People’s attitude towards wildlife conservation

**Figure 4:**
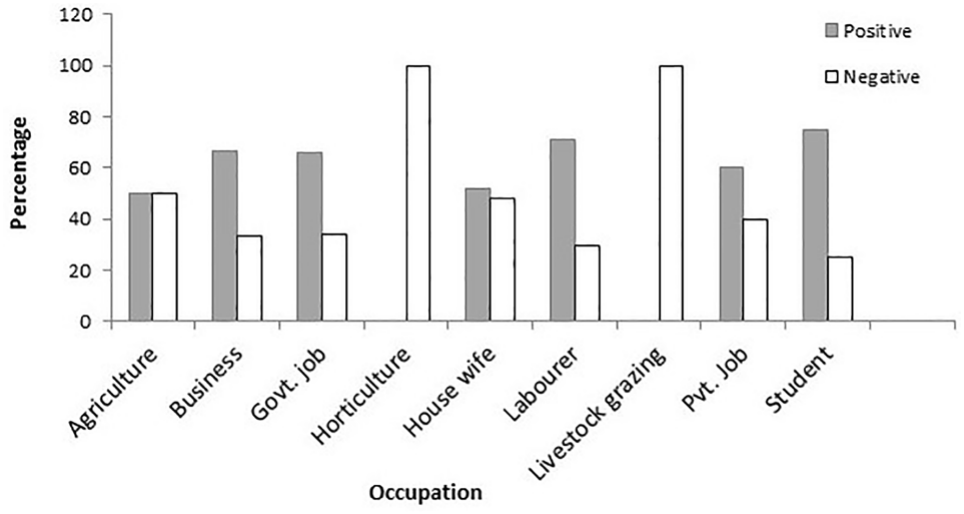
Attitude of people involved in different occupations around the forest corridor linking the Rajaji and Corbett Tiger Reserves, Uttarakhand, India.

In spite of HWC, 68.8% of respondents showed a positive attitude towards wildlife conservation, while 15.7% had a negative attitude, and the rest (15.5%) were indifferent. Higher percentage of male respondents (77.3%), people living farther (> 2 km) from the periphery of the forest corridor (76%), people living in the plains (61.9%), old people (59.1%), people not involved in agriculture (59.6%), people without livestock (57.5%) and people not extracting resources from the forest (67.7%) exhibited a positive attitude towards wildlife conservation (Fig. 4). The likelihood that a person will show a positive attitude towards wildlife conservation decreased if he or she resided in the hills and if there was more wildlife (Table 4).

### Payments to encourage coexistence (PEC)

The annual accounting profit from cultivating a hectare of land, in the absence of crop depredation by wild animals, was estimated at US $ 3571.84 ha^−1^ for the plains and US $ 361.44 ha^−1^ for the hills. The net present value (NPV) of the benefits to the local communities from participating in the PEC programme for 5 years, discounted at 12%, is US $ 12,875.7 ha^−1^ and US $ 1302.9 ha^−1^, for the plains and the hills, respectively, which is the profit from not cultivating a hectare of land (Table 5). The accounting profit and NPV is calculated on a per-hectare basis; therefore, the amount payable will be proportionate to the land cultivated by a particular household. The incentives are proposed to be paid to the farmers for the land on which they abstain from all forms of agricultural practices for a duration of 5 years.

**Table 5:** Net present value of the benefits to the local communities from participating in the payments to encourage coexistence programme for 5 years.

Discount rate (%)	Plains (US $ ha^−1^)	Hills (US $ ha^−1^)
0	17,859.25	1807.25
5	15,464.2	1564.9
10	13,540.15	1370.2
12	12,875.7	1302.9
15	11,973.4	1211.6

@ 2014 prices.

The profit from not cultivating the land and participating in the programme will be greater than the profit from cultivating, since the payment made to the farmers will cover the costs incurred due to crop damage as the estimated NPV is the profit earned without crop depredation by wild animals. Furthermore, there are hidden costs of HWC, such as increased food insecurity, risk of injury and death from guarding, psychological and social costs^20,29,30^, which can be avoided under the proposed programme.

## Discussion

The Terai-Arc Landscape (TAL) in the foothills of the Himalaya, is among the 200 globally important eco-regions in the world^31^. It’s a transboundary landscape encompassing an area of 49,500 km^2^ between River Yamuna in the west and River Bhagmati in the east, in India and Nepal, respectively. Around 15 PAs exist in the landscape including five TRs viz., Rajaji, Corbett, Pilibhit, Dudhwa and Valmiki (west to east), in the Indian part of the landscape. This landscape provides shelter to several species of conservation concern viz., tiger (*Panthera tigris*), leopard (*Panthera leo*), sloth bear (*Melursus ursinus*), Himalayan black bear (*Ursus thibetanus*), Asian elephant (*Elaphus maximus*), greater one-horned rhinoceros (*Rhinoceros unicornis*), Himalayan serow (*Capricornis sumatraensis*) and hog deer (*Axis porcinus*). The Rajaji and Corbett TRs in the western Himalayan foothills support a tiger population that is genetically distinct from the tigers in Valmiki TR, located in the eastern region of the landscape^32^. Apart from tiger, this region supports a population of ~ 2040 elephants distributed in six isolated populations^12,33^. The Rajaji–Corbett forest corridor is vital for the free movement and exchange of genetic material between the western and eastern part of the foothills in India and Nepal. Additionally, this forest corridor provides an array of ecosystem services integral to the wellbeing of local communities^7^. Thus, intactness of the corridor is crucial for maintaining the gene pool and for the wellbeing of the local communities residing in this landscape.

For the rural communities residing in and around this corridor, the forest has remained a major source of fuelwood, fodder and food, for both sustenance and income generation^6^. The overall extraction of forest resources has increased in comparison with the findings of an earlier study around the corridor^6^. This could be attributed to the increase in human population in the area, since the consumption of forest biomass per household has reduced due to the availability of alternative energy sources, namely LPG and kerosene. A large proportion of households (70%) in the hills is still dependent on the forest for fuelwood, perhaps due to habitual or traditional dependence on the forest for biomass^6^ combined with lack of alternatives or difficulty in accessing modern and efficient resources. The contribution of forest resources to the household income was greater in the hills than in the plains.

Our study showed that HWC is a serious issue around this forest corridor. Among the crop raiders, Asian elephants and wild pigs were reported as the main problematic species in the area affecting the household income considerably. In almost all the elephant-range countries where elephants survive in fragmented and disturbed habitats, crop raiding has been reported^34–37^. Crop raiding by animals may be due to a higher sugar content, and lower fibre and secondary defensive chemicals content in crops compared to their wild counterparts^38^. Elephants have a natural preference for derivatives of plants from the family Gramineae, including wheat and rice^39^. Moreover, wild pigs can dig burrows and screen themselves from guards in crops such as sugarcane and maize, which grow to heights of more than 2 m^40^.

Most of the respondents reported that the cases of livestock depredation occurred in the forest while grazing. The attacks on livestock may also be due to the high density of livestock around the forest corridor. Sangay and Vernes^41^ also identified leopard as the major predator of livestock in human-dominated landscapes. Leopards preyed more on calves, cows and goats compared to other livestock. Patterson et al.^42^ speculated that body size and availability of prey species govern the selection of prey species. Leopards, weighing 30**–**40 kg, take prey in the weight range of 25**–**50 kg^43^; leopards in India take prey generally weighing less than 50 kg^44^. Our results match the trends of these studies, which were conducted in the wild. Calves, being small and weighing less than 25 kg, may be easy prey for leopards in the forest. Human injuries or deaths mostly occurred as a result of chance encounters with elephants or leopards.

Our results mirror findings of other studies^45–47^ where likelihood of crop damage was negatively associated with the distance from the forest, and positively associated with cultivation of wheat, sugarcane and pulses, and number of crops cultivated. Likelihood of livestock loss was positively associated with the presence of calves and indigenous buffaloes. We found that crop loss and livestock loss are functions of multiple factors^45–47^.

People residing in plains supported wildlife conservation, similar to the findings of Badola^7^, which may be credited to the high literacy rate and availability of alternative livelihoods. However, the people in the hills were not very positive towards wildlife conservation, which may be due to their higher dependence on agriculture and some recent and frequent incidents of crop damage and livestock attacks. Our results revealed that support for wildlife conservation by the people decreased with increase in wildlife population.

The positive attitude of the people towards wildlife conservation might alter in the future when the continuous and increased losses to their crops and livestock become excessive, which may be calamitous to the conservation of the corridor. Physical barriers like electric fencing and brick or stone walls have proven to be ineffective in the study area, since the electric fence and stone or brick walls were broken by elephants, and the local communities were unable to maintain it as it was physically, socially and economically taxing. Additionally, guarding crops against elephants has a high risk of human injury or casualty. Compensations offered for damage incurred are insufficient and subject to processing delays and corruption^18^. Furthermore, the people suffering losses due to HWC refrain from filing the claim due to the high transaction cost (physical, economic and social) that is incurred in the form of documentation, multiple visits to government offices, procedural delays etc.^48^. Insurance schemes are subject to issues of moral hazard, adverse selection and fraudulent claims due to difficulties associated with verification of damage and investment in damage avoidance^49^. Given the socioeconomic profile of the local communities, there is a high risk of running out of pooled funds in case of multiple claims, making micro insurance schemes unviable. An effective and sustainable HWC mitigation strategy must address issues of both biodiversity conservation and poverty alleviation.

The proposed incentive-based mitigation strategy viz., payments to encourage coexistence (PEC), is based on the principles of PES wherein the custodians of an ecosystem are paid by the beneficiaries of the ecosystem services, and the payment is conditional on the supply of the ecosystem services or the adoption of certain management practices that are subject to monitoring under the terms of the contract. PES has been implemented across the globe to incentivize landowners to encourage land management practices that promote environment conservation and enhance the flow of select ecosystem services, with varying degrees of success. Grima et al.^50^ analysed the performance of 40 PES programmes associated with provision of water in Latin America, and classified 23 cases as successful and 12 as partially successful. The Produtor de Água and Conservador das Água watershed PES programmes, implemented in the Brazilian Atlantic forest to improve water quality and quantity in the Cantareira water supply system through soil and water conservation activities, has resulted in an additional 2.8–5.6% of farm area coverage under Atlantic forest over a 5-year period through forest regeneration^51^. The ‘Grain for Green’ programme has led to the establishment of 29.1 × 10^6^ ha of forest in 25 provinces in China through conservation of natural forest, afforestation of farmland and degraded land, and establishment of fruit tree plantations^52^. Tuanmu et al.^53^ examined the impact of the Natural Forest Conservation Program in China on giant panda habitat in Wolong Nature Reserve and noted that areas monitored by local residents showed greater improvement in habitat compared to areas monitored by the local government, provided they are adequately paid.

Under the proposed PEC programme, financial assistance will be given to local communities to encourage coexistence with wildlife by mitigating HWC through behaviour change and opportunities to explore alternate livelihoods, in order to ensure the functionality of the forest corridor. The intended beneficiaries are the farmers who will be paid for not cultivating on their land for a trial period of 5 years; thus, tackling crop depredation by wild animals. The farmers will be encouraged to enter into contract with the herders, under which their livestock can graze on the now uncultivated land for a mutually agreed upon amount. This will mitigate livestock depredation by reducing the entry of cattle into forests, where majority of the incidents of livestock depredation were reported. The PEC programme not only addresses the visible costs of HWC but also the invisible costs, and avoids the distress associated with relocation.

The proposed incentive mechanism has elements of both compensation schemes and alternate livelihood strategy for HWC mitigation. Since the farmers in the region are mainly marginal farmers practicing subsistence agriculture, coupled with the fact that income from off-farm sources was considerably higher than from crop cultivation, the proposed PEC programme will increase the income of participating households not only through direct monetary transfer, but also through promotion of off-farm labour participation by relaxing liquidity constraints. An increase in off-farm labour participation was noted in China as a result of the ‘Grain for Green’ programme, especially among the poor, young and more educated sections of the participating households^54^. Wildlife-based tourism is a possible off-farm source of livelihood that can be explored by the local communities residing in the vicinity of the forest corridor; since, the region has immense potential for wildlife-based tourism given its close proximity to two PAs viz., Rajaji and Corbett TRs. With the development of appropriate tourism infrastructure in the region, backed by the Government of Uttarakhand’s tourism policy initiatives, wildlife-based tourism can be a lucrative business and add to the capital stock of the participating households. The wildlife tourism-based PES programme implemented in the Olare Orok Conservancy in Maasai Mara, Kenya, wherein pastoral landowners were voluntarily relocated and asked to stop livestock grazing inside the conservancy (reserved for wildlife tourism) in exchange for direct monetary payments by private tourism operators, resulted in an increase in the physical, human and social capital of participating households, through cash income diversification, creation of employment opportunities in the conservancy, and prevention of poverty by serving as a buffer during climate shocks^55^.

Barriers to the adoption of such programmes include a lack of awareness among the farmers, mistrust of the forest department and lack of funding^56^. Custodians may also not appreciate the importance of the programme’s conditionality, which in the present case is the need to refrain from agriculture to continue receiving the payments. Such obstacles, however, are common to all incentive-based schemes^57^. The PEC programme must be supported with a targeted public outreach campaign, establishment of linkages with the concerned Government departments and agencies, and capacity building of the intended beneficiaries to enable them to access the benefits of the programme. In order to ensure compliance, the study area must be monitored by the Uttarakhand Forest Department, under whom the management of the forest falls, including an annual evaluation of the programme. In order to improve the efficacy of the PEC programme, monitoring duties should also be assigned to locals, from both participating and non-participating households, because an incentive-based strategy that complements not only a command-and-control strategy, but also community- and norm-based strategies has the potential to achieve a higher degree of conservation effectiveness and HWC mitigation^53^. In the Periyar TR, India, an all-women forest patrolling team, known as the “*Vasantha Sena*”, voluntarily patrol the forest in groups of six to eight during the day, from 11 a.m. to 5 p.m., with their presence serving as a deterrent to illegal entry and biomass extraction^58^. The centrally sponsored ‘Integrated Development of Wildlife Habitats’ scheme that provides assistance to State/Union Territories for the conservation of wildlife and its habitats, inside and outside protected areas, can be a source of funding for the proposed programme^59^. Sustainable, timely and continuous economic benefits accruing to the community will change their attitude and increase the tolerance exhibited towards wildlife^60,61^. Furthermore, these measures should be combined with additional interventions in the form of policy actions in other sectors that are aimed at reversing the current dynamics of crop intensification and urban encroachment; since, agriculture in the region has become more commercial, and agricultural land has been converted into commercial or residential areas^33^.

The proposed incentive-based mitigation strategy is a pragmatic and practical means to engage local communities in wildlife conservation, which is a major challenge in heavily populated developing countries like India^62^. Due to the replicability and scalability of the model, the proposed PEC programme can be implemented in the fringes of PAs and wildlife corridors, in order to create a larger model of community engagement for biodiversity conservation that views preservation and protection of crucial wildlife habitat at the landscape level, rather than in pockets.

## Materials and methods

### Study area

**Figure 5:**
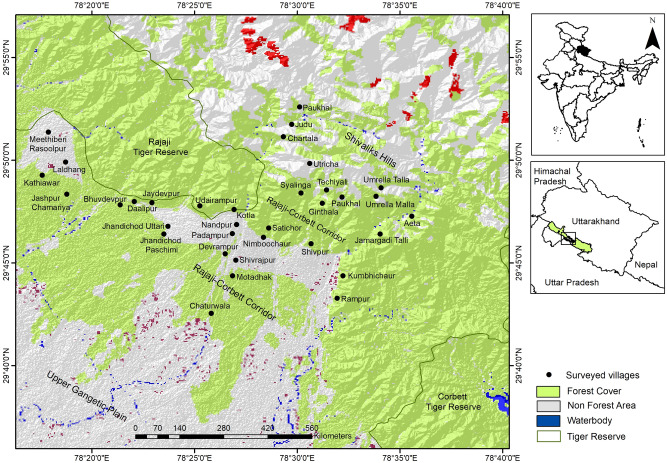
Map highlighting the forest corridor linking the Rajaji and Corbett Tiger Reserves, and the locations of the sampled villages in Uttarakhand, India. Land cover used was downloaded from Diva-Gis (http://www.diva-gis.org/gdata). The map was created using ArcGIS v.10.3.1 software developed by ESRI (https://www.esri.com).

The forest corridor linking the Rajaji and Corbett TRs (29° 37′ to 29° 53′ N and 78° 19′ to 78° 41′ E), located in the Indian state of Uttarakhand (Fig. 5), comprises of two smaller corridors—one through the Shivalik Hills (Lansdowne Forest Division) and other through the forests of the Shivalik foothills (Haridwar and Bijnore Forest Divisions). Due to its location along the foothills of the Himalayas, the topography is hilly, with innumerable spurs and high ridges in the north. The southern portion of the corridor is characterized by the Bhabar tract, which gradually merges with the Gangetic plains. The area has a sub-tropical climate, with temperatures ranging between 7 and 42 °C and annual rainfall ranging between 1253 and 2053 mm, with the maximum rainfall received between March and June. The tract is drained by numerous rivers and streams running from north to south, most of which are dry in late winter and summer.

The vegetation consists of northern tropical moist and dry deciduous forests with species such as *Shorea robusta, Mallotus philippensis, Kydia calycina, Dalbergia sissoo, Acacia catechu, Ougeinia oojeinensis* and *Terminalia* spp. The Rajaji–Corbett forest corridor maintains the genetic diversity of large mammals such as tiger (*Panthera tigris*), leopard (*Panthera pardus*), Asian elephant (*Elephas maximus*), sloth bear (*Melursus ursinus*), Himalayan black bear (*Ursus thibetanus*), sambar (*Rusa unicolor*) and others^11^. An increase in the number of human settlements, coupled with agricultural expansion and commercial resource extraction from the corridor, have reduced the area that was once available to wildlife^11,63^. Geographically, the villages in the area can be divided into two groups viz., the northern hill villages and the southern plains villages. Terrace farming is prevalent in the northern hilly region, where mostly women are the heads of households because the male members migrate to the plains in search of employment. The southern villages are economically better off due to their nearness to the urban centre of Kotdwar, productive agricultural land and more employment opportunities in industries along the fringes of the town^6^. The major communities inhabiting this area include (1) *Garhwalis*, hill residents employed in both marginal agriculture and private jobs, (2) *Boksas*, a forest-dependent tribe who work as daily labourers in nearby towns, (3) *Gujjars*, the transhumance pastoralists living inside the forest and engaged in cattle rearing, and (4) *Bhotias*, migratory grazers of Indo-Tibetan origin who visit the area in winter with herds of goats and sheep.

### Methodology

Villages within 5 km from the forest corridor were identified using a digitized map of the study area. A total of 205 villages were identified, and data on the location and distribution of the villages (distance from the forest corridor), demographic profile, accessibility by motorable roads from urban areas, livestock owned, dependence on forest resources (timber, non-timber forest products like fuelwood, fodder etc.) and accessibility to basic facilities such as primary health centres, schools, transportation and alternative fuel resources like LPG was collected in the rapid rural appraisal format^6,28^. Village level data was also collected from various government and non-government organizations. This village level information was subjected to principal component analysis as in Lee et al.^64^ and Badola et al.^28^, and results were used to perform hierarchical cluster analysis, which divided the villages into three clusters. Representative villages from each cluster were selected.

A total of 757 households from 36 villages were surveyed and personal interviews with house representatives were conducted using a semi-structured questionnaire with both open- and close-ended questions, between May 2013 and December 2014. Each interview lasted for about 45 minutes to 1 hour. Information about crop raiding, livestock depredation (2012–2014) and human injuries/deaths caused by wildlife, as well as dependence on the forest, occupational structure, agricultural holdings and livestock owned was gathered. Conflict locations in case of livestock depredation and crop raiding were visited to validate the data pertaining to damage.

### Ethics statement

The questionnaires and social surveys conducted in this study were duly approved by the institutional review board (Training Research and Advisory Committee) of the Wildlife Institute of India (WII), Dehradun, India, and were conducted in accordance with the guidelines and regulations of WII. Informed consent was obtained from the heads of the community and the respondents who participated in this survey. Prior to interviews, respondents were informed about the objectives of the survey and nature of questions. Respondents were asked if the information provided by them can be used for any report or article in any form. Informed consent of the respondents was also obtained to publish the obtained information/image(s) in an online open access publication. The study did not involve experiments using any live or dead animal or human subjects and therefore, did not require the approval of the animal ethics committee.

### Data analysis

The data pertaining to socio-economic profile, conflict and attitude of local community was summarised in the form of frequencies, proportions and measures of central tendency. The monetary benefit and loss per household was calculated by multiplying with the market price of various forest resources extracted, and livestock and crops lost. The economic loss resulting from deaths/injuries caused by wildlife was calculated by aggregating the money spent on treatment and income lost due to injuries.

### Factors governing human–wildlife conflict

Self-reported incidents of crop damage (n = 368) were modelled as a function of environmental factors (distance from the forest), agricultural factors (number of crop types grown in a year, crop types cultivated, size of the landholding reported), demographic characteristics (family size, gender of the respondent, occupation) and use of mitigation measures. Logistic regression with the forward conditional method was used in the modelling. Similarly, livestock depredation (2012–2014) was modelled as a function of the distance from the forest and the number and type of livestock owned (Table S3). The variables were tested for multi-collinearity before the logistic regression was run. All the analyses were conducted using SPSS 16.0.

### Payments to encourage coexistence

The incentive mechanism designed here is based on the concept of PES, which is the best-suited approach for getting the support of local communities for conservation^24^. Conservation payments involving HWC have been termed as “payments to encourage coexistence” (PEC) as such payments do not always follow the strict criteria of PES although the overall concept complies with any PES programme^24^.

Profit, defined in economic theory as the difference between the revenue earned and the cost incurred in the production, is a function of the quantity produced, the price of the commodity produced and the cost of the inputs^65^. Since the area is close to the forest, the fields are often raided by wild animals. This affects the quantity produced and thus affects the profits. Hence, the profit earned from cultivating a particular piece of land can be defined as:

$${\prod }_{c}=\prod \{{Y}_{A}\left[{Q}_{A}\left(I,CD\right), {P}_{A}\right], {C}_{I}\}$$

where, ${\prod }_{c}$ is the profit earned from cultivating the land, Y_A_ is the income generated from cultivating the land, Q_A_ is the quantity of agricultural produce, I is the inputs used, CD is the crop damage due to wildlife, P_A_ is the price of agricultural produce and C_I_ is the cost of inputs used.

To accept a payment mechanism to abstain from cultivating a particular plot of land, the profit generated from not cultivating must be greater than or at least equal to the profit generated from cultivating that plot of land. The financial incentive should be designed such that

$$\prod_{NC} \ge \prod_{C}$$

where, ${\prod }_{NC}$ is the profit from not cultivating the land.

In the current study, we estimated the annual payment to be paid to the local communities for not cultivating in the conflict prone areas by calculating the accounting profit from cultivating a hectare of land in the absence of crop depredation by wild animals, for the year in which the study was undertaken. Since, we are interested in a programme that will show a positive outcome over a long period i.e., reduced HWC, the programme must be implemented for at least 5 years^21^.

In order to estimate the benefits to the local communities from participating in the programme, the NPV of the annual payment over 5 years was estimated, assuming no decline in the purchasing power of the Indian rupee over the 5-year period and keeping the cash flow constant at the estimated accounting profit. NPV is considered ideal for determining the benefits accrued to the local communities as it accounts for the time preference of the communities, represented by the discount rate^66^. A social discount rate of 12% has been set for projects and public investments in India^67^. Due to a lack of consensus and the policy implications of using such fixed discount rates, we discounted the NPV using different discount rates to reflect the different time preferences of the people. Higher the discount rate, higher is the presumed time preference for immediate costs and benefits, and lower is the value placed on future benefits and costs.

The NPV is the present value of the expected net benefit over time. The NPV was estimated using the following equation:

$${\text{NPV}} = \mathop \sum \limits_{n = 1}^{5} \left[ {\frac{{Y_{A} - C_{I} }}{{\left( {1 + i} \right)^{n} }}} \right],$$

where, Y_A_ is the income generated from cultivating the land, C_I_ is the cost of inputs used, i is the discount rate, n is the number of years (the duration of the trial period i.e., 5 years).

Under the proposed incentive mechanism, profit from not cultivating the concerned piece of land is defined as:

$$\prod_{NC} = {\text{NPV}}{.}$$

## Supplementary Information

Supplementary Information.
