# Supplementary material for: An incentive-based mitigation strategy to encourage coexistence of large mammals and humans along the foothills of Indian Western Himalayas
Source: Sci Rep. 2021 Mar 4;11:5235. doi: 10.1038/s41598-021-84119-7 (PMC7933403; doi:10.1038/s41598-021-84119-7)
Supplement: Supplementary file 1 — Supplementary Information. [file 41598_2021_84119_MOESM1_ESM.docx]

**Supplementary information**

An incentive-based mitigation strategy to encourage coexistence of large mammals and humans along the foothills of Indian Western Himalayas

Ruchi Badola^1^, Tanveer Ahmed^1^, Amanat Kaur Gill^1^, Pariva Dobriyal^1^, Goura Chandra Das^1^, Srishti Badola^1^, Syed Ainul Hussain^1^*

^1^Wildlife Institute of India, Chandrabani, Post Box # 18, Dehradun (248001), Uttarakhand, India

*Corresponding author:

Email: [hussain@wii.gov.in](mailto:hussain@wii.gov.in) (SAH)

**Table S1.** Percentage of households dependent on various sources for fuel around the forest corridor linking the Rajaji and Corbett Tiger Reserves, Uttarakhand, India.

| **Sources of fuel** | **Hill** | **Plain** | **Overall** |
| --- | --- | --- | --- |
| Wood | 13.71 | 22.50 | 20.21 |
| LPG + Wood + Biogas + Solar cooker | 0.51 | 0.00 | 0.13 |
| LPG + Dung + Wood | 0.51 | 1.25 | 1.06 |
| Biogas | 0.00 | 0.18 | 0.13 |
| Kerosene + Dung + Wood | 0.51 | 0.18 | 0.26 |
| Dung + Wood | 0.51 | 0.18 | 0.26 |
| LPG + Dung + Wood + Biogas | 0.00 | 0.18 | 0.13 |
| LPG + Wood | 40.61 | 54.82 | 51.12 |
| LPG + Bio gas | 0.00 | 0.18 | 0.13 |
| LPG | 3.05 | 11.43 | 9.25 |
| Kerosene + Wood | 27.92 | 4.29 | 10.44 |
| LPG + Kerosene + Wood | 10.66 | 0.89 | 3.43 |
| LPG + Wood + Biogas | 1.02 | 2.68 | 2.25 |
| Kerosene + Wood + Biogas | 0.51 | 0.36 | 0.40 |
| Wood + Biogas | 0.51 | 0.89 | 0.79 |

**Table S2.** Percentage of households engaged in different combinations of livelihood option around the forest corridor linking the Rajaji and Corbett Tiger Reserves, Uttarakhand, India.

| **Combination of livelihood options** | **Percentage of households** |
| --- | --- |
| Agriculture | 15.6 |
| Agriculture + Business | 1.3 |
| Agriculture + Govt. job | 1.7 |
| Agriculture + Pvt. job | 11.1 |
| Agriculture + Pvt. job+ Govt. job | 1.1 |
| Agriculture + Retd. Govt. job | 0.5 |
| Agriculture + Retd. Govt. job + Pvt. job | 0.7 |
| Retd. Govt. job + Govt. job | 1.1 |
| Retd. Govt. job + Business | 0.5 |
| Labourer | 12.2 |
| Govt. job | 5.0 |
| Business | 4.9 |
| Pvt. job | 9.0 |
| Retd. Govt. job | 3.3 |
| Retd. Govt. job + Pvt. job | 3.4 |
| Agriculture + Labourer | 1.6 |
| Business + Pvt. job | 2.8 |
| Horticulture + Business + Pvt. job | 0.1 |
| Govt. job + Pvt. job | 2.1 |
| Pvt. job + Labourer | 3.7 |
| Govt. job + Pvt. job + Business | 0.5 |
| Business + labourer | 0.4 |
| Retd. Govt. job + Business + labourer | 0.1 |
| Retd. Govt. job + govt. job + Pvt. job | 0.8 |
| Business + labourer + Govt. job | 0.1 |
| Agriculture + Pvt. job + Business | 0.5 |
| Govt. job + Labourer | 0.8 |
| Agriculture + Business + Govt. job | 0.3 |
| Retd. Govt. job + Pvt. job + Business | 0.4 |
| Unemployed | 1.8 |
| Business + Unemployed | 0.3 |
| Retd. Govt. job + Govt. job + Unemployed | 0.3 |
| Govt. job + Pvt. job + Unemployed | 1.6 |
| Agriculture + Unemployed | 0.7 |
| Pvt. job + Unemployed | 1.3 |
| Agriculture + Pvt. job + Unemployed | 0.4 |
| Labourer + Unemployed + Business | 0.3 |
| Agriculture + Labourer + Pvt. job | 0.5 |
| Govt. job + Pvt. job + Labourer | 0.4 |
| Retd. Govt. job + Govt. job + Pvt. job + Unemployed | 0.3 |
| Agriculture + Govt. job + Pvt. job + Labourer | 0.1 |
| Labourer + Unemployed | 1.3 |
| Retd. Govt. job + Govt. job + Pvt. job + Business | 0.3 |
| Retd. Govt. job + Pvt. job + labourer | 0.4 |
| Pvt. job + Business + labourer | 0.1 |
| Agriculture + Retd. Govt. job + Govt. job | 0.1 |
| Livestock | 0.1 |
| Agriculture + Retd. Govt. job + Labourer | 0.3 |
| Retd. Govt. job + Labourer | 0.1 |
| Pvt. job + labourer + Unemployed | 0.4 |
| Govt. job + Business | 0.1 |
| Govt. job + Unemployed | 1.3 |
| Govt. job + Labourer + Unemployed | 0.5 |
| Govt. job + Pvt. job + Business + Unemployed | 0.1 |
| Pvt. job + Business + Unemployed | 0.3 |
| Agriculture + Pvt. job + Business + Unemployed | 0.1 |
| Govt. job + Business + Unemployed | 0.3 |
| Retd. Govt. job + Pvt. job + Unemployed | 0.3 |
| Agriculture + Govt. job + Unemployed | 0.1 |
| Retd. Govt. job + labourer + Unemployed | 0.1 |

Pvt. – Private; Retd. - Retired

**Table S3.** Details of variables collected from surveys and used in logistic regression.

| **Variables** | **Crop Damage** | **Livestock Depredation** | **Attitude** |
| --- | --- | --- | --- |
| Distance to forest | √ | √ | √ |
| Land holding | √ | * | * |
| Rice | √ | * | * |
| Wheat | √ | * | * |
| Sorghum | √ | * | * |
| Manduwa | √ | * | * |
| Brown mustard seeds | √ | * | * |
| Grass | √ | * | * |
| Maize | √ | * | * |
| Gaith | √ | * | * |
| Jhingur | √ | * | * |
| Sugarcane | √ | * | * |
| Vegetables | √ | * | * |
| Fruits | √ | * | * |
| Spices | √ | * | * |
| Til | √ | * | * |
| Total crops | √ | * | * |
| Gender | √ | * | * |
| Indigenous Cow | * | √ | * |
| Hybrid cow | * | √ | * |
| Indigenous Buffalo | * | √ | * |
| Hybrid buffalo | * | √ | * |
| Goat | * | √ | * |
| Sheep | * | √ | * |
| Poultry | * | √ | * |
| Ox | * | √ | * |
| Calf | * | √ | * |
| Age of respondent | * | * | √ |
| Occupation | * | * | √ |
| Total family members | * | * | √ |
| Agriculture practice | * | * | √ |
| Livestock | * | * | √ |
| Benefit from forest | * | * | √ |
| Increase in wildlife | * | * | √ |

√ = Used, *= not used in the logistic regression

**Table S4.** Destruction of various crops by animals around the forest corridor linking the Rajaji and Corbett Tiger Reserves, Uttarakhand, India.

| **Crop type** | **Area (ha hh^-1^ year^-1^)** | **Quantity (kg year^-1^)** |
| --- | --- | --- |
| Wheat | 0.09 ± 0.02 | 57.24 ± 9.9 |
| Rice | 0.44 ± 0.02 | 804.59 ± 48.2 |
| Sugarcane | 0.004 ± 0.00 | 83.04 ± 22.31 |
| Vegetables | 0.03 ± 0.01 | 93 ± 24.4 |
| Spice | 0.01 ± 0.02 | 23.26 ± 14.23 |
| Pulses | 0.04 ± 0.02 | 8.92 ± 8.92 |


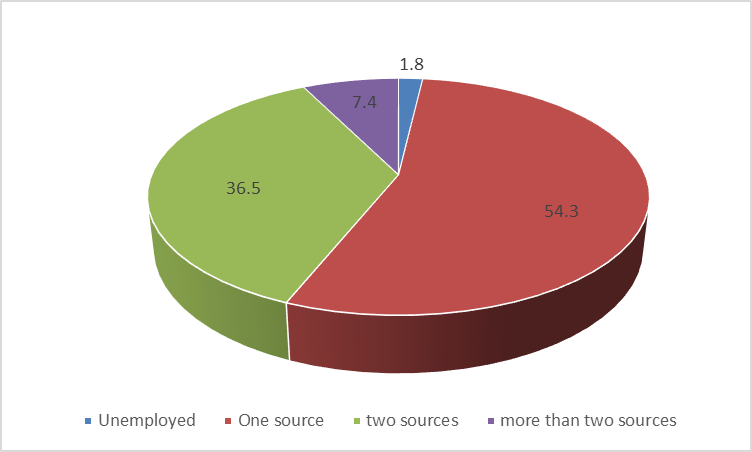


**Figure S1.** Percentage of household dependent of number of livelihood option to earn their livelihood around the forest corridor linking the Rajaji and Corbett Tiger Reserves, Uttarakhand, India.


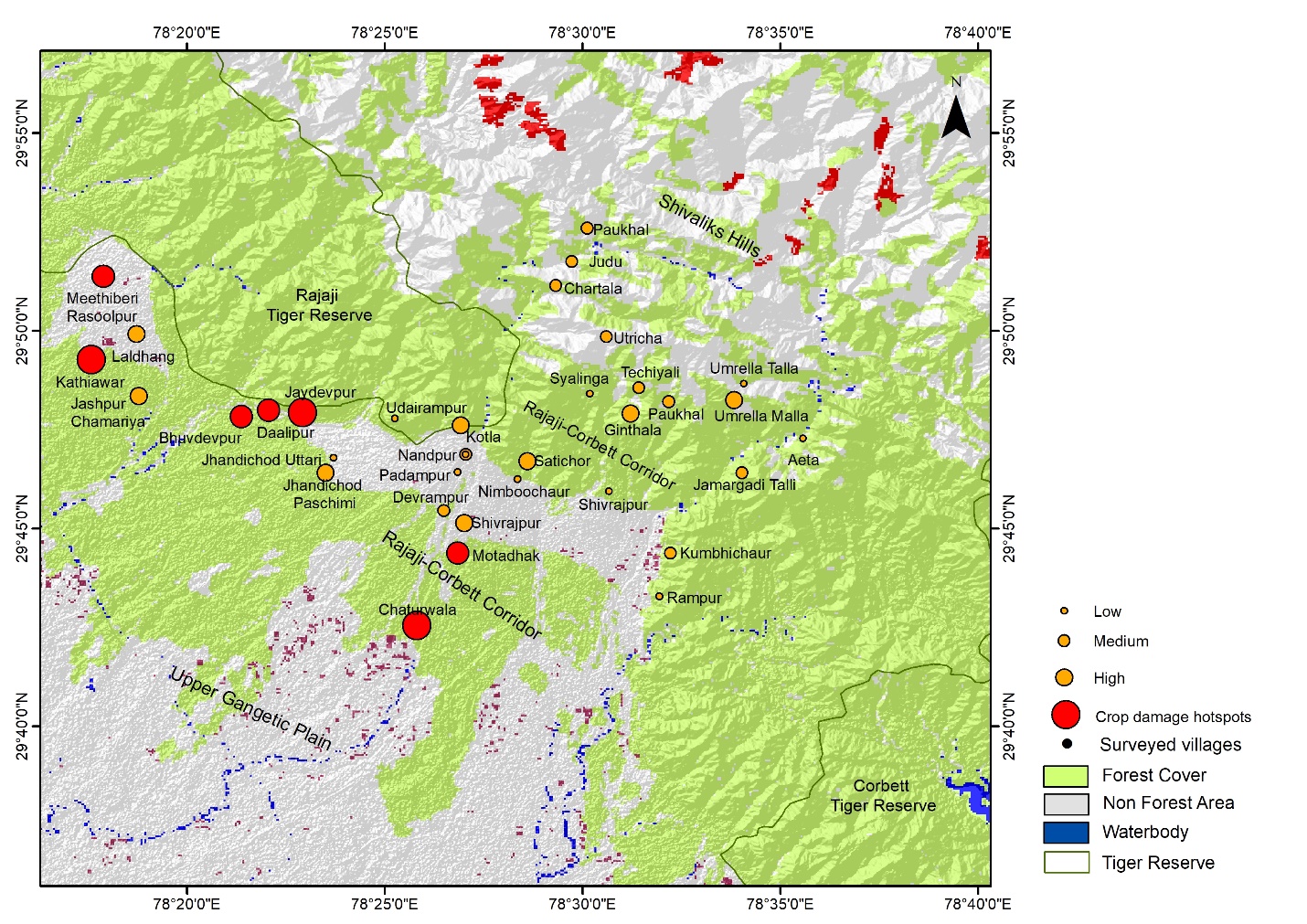


**Figure S2.** Villages identified as crop raiding hotspots around the forest corridor linking the Rajaji and Corbett Tiger Reserves, Uttarakhand, India, on the basis of average cost incurred (US$ hh^-1^ year^-1^) due to crop raiding. Land cover used was downloaded from Diva-Gis (<http://www.diva-gis.org/gdata>). The map was created using ArcGIS v.10.3.1 software developed by ESRI (https://www.esri.com).


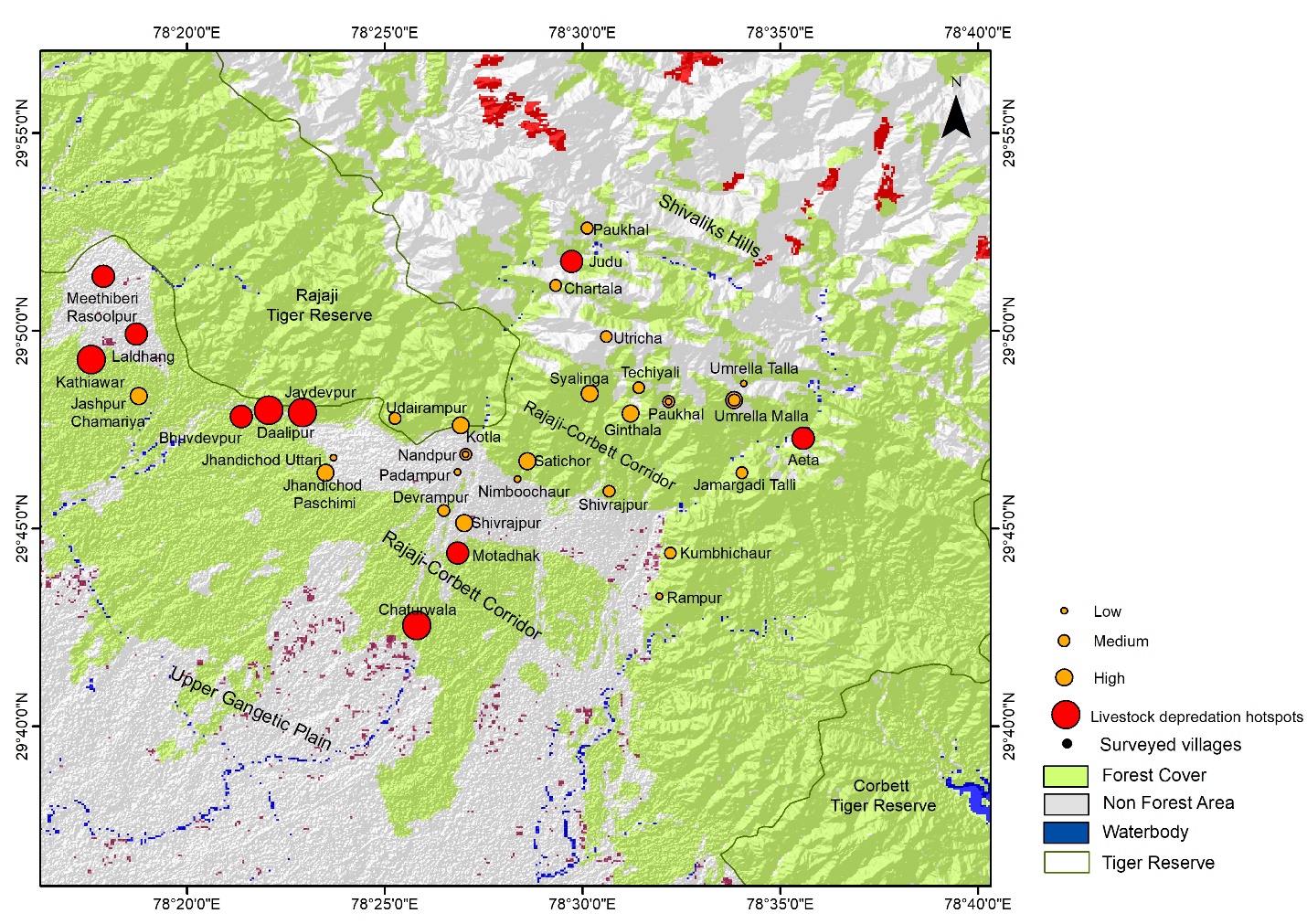


**Figure S3.** Villages identified as livestock depredation hotspots around the forest corridor linking the Rajaji and Corbett Tiger Reserves, Uttarakhand, India, on the basis of average cost incurred (US$ hh^-1^ year^-1^) due livestock depredation. Land cover used was downloaded from Diva-Gis (<http://www.diva-gis.org/gdata>). The map was created using ArcGIS v.10.3.1 software developed by ESRI (https://www.esri.com).

.
